# Tobacco Root Microbial Community Composition Significantly Associated With Root-Knot Nematode Infections: Dynamic Changes in Microbiota and Growth Stage

**DOI:** 10.3389/fmicb.2022.807057

**Published:** 2022-02-09

**Authors:** Yi Cao, Zhi-Xiao Yang, Dong-Mei Yang, Ning Lu, Shi-Zhou Yu, Jian-Yu Meng, Xing-Jiang Chen

**Affiliations:** Guizhou Academy of Tobacco Science, Guiyang, China

**Keywords:** root-knot nematode, microbiota, microbial community, root, time dynamic

## Abstract

The root-knot nematode (RKN) is an important pathogen that affects the growth of many crops. Exploring the interaction of biocontrol bacteria-pathogens-host root microbes is the theoretical basis for improving colonization and controlling the effect of biocontrol bacteria in the rhizosphere. Therefore, 16S and 18S rRNA sequencing technology was used to explore the microbial composition and diversity of tobacco roots (rhizosphere and endophytic) at different growth stages in typical tobacco RKN-infected areas for 2 consecutive years. We observed that RKN infection changed the α-diversity and microbial composition of root microorganisms and drove the transformation of microorganisms from bacteria to fungi. The abundance of *Sphingomonas* decreased significantly from 18% to less than 3%, while the abundance of *Rhizobiaceae* increased from 4 to 15% at the early growth stage during the first planting year, and it promoted the proliferation of *Chryseobacterium* at the late growth stage in rhizosphere microorganisms with the highest abundance of 17%. The overall trend of rhizosphere microorganisms changed in the early growth stage with increasing growth time. The specific results were as follows: (1) *Rhizobiaceae* and *Chryseobacterium* increased rapidly after 75 days, became the main abundant bacteria in the rhizosphere microorganisms. (2) The dominant flora in fungi were *Fusarium* and *Setophoma*. (3) Comparing the root microbes in 2017 and 2018, RKN infection significantly promoted the proliferation of *Pseudomonas* and *Setophoma* in both the rhizosphere and endophytes during the second year of continuous tobacco planting, increasing the relative abundance of *Pseudomonas* from 2 to 25%. *Pseudomonas* was determined to play an important role in plant pest control. Finally, a total of 32 strains of growth-promoting bacteria were screened from tobacco rhizosphere bacteria infected with RKN through a combination of 16S rRNA sequencing and life-promoting tests. The results of this research are helpful for analyzing the relationship between RKNs and bacteria in plants, providing reference data for elucidating the pathogenesis of RKNs and new ideas for the biological control of RKNs.

**GRAPHICAL ABSTRACT:**
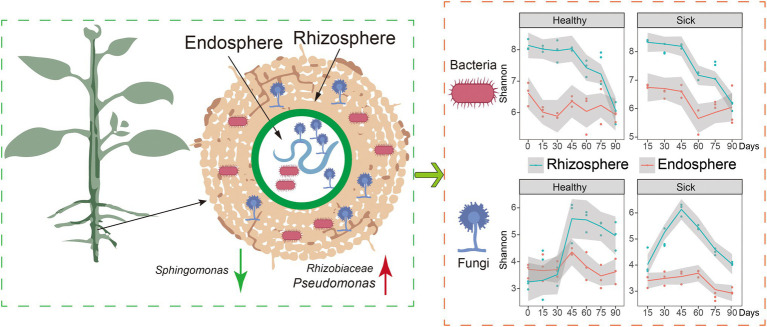

## Introduction

Root-knot nematode (RKN) (*Meloidogyne* spp.) disease is an important crop disease that has become one of the main factors limiting sustainable crop production (Gorny et al., 2019; Hemmati and Saeedizadeh, 2020). RKN infects plant roots and forms nodules, which hinder the absorption of nutrients and water by the roots, leading to wilting and withering; in addition, RKN infection reduces plant resistance, causes wounds, is conducive to infection by other pathogens, and often causes complex diseases (EFSA Panel on Plant Health et al., 2018; Khan and Ahamad, 2020), such as Fusarium wilt, root rot, and bacterial diseases (Khan and Ahamad, 2020; Leonetti and Molinari, 2020). With the prohibition of methyl bromide and the appearance of chemical nematicides with high toxicity and high residue, such as imidacloprid, aldicarb, and carbofuran, there are few nematicides that both have the control effect and are safe for use in agriculture (Huang et al., 2016). Therefore, due to the lack of effective resistant varieties, the high multiple cropping index of crops, the limitation of the use of chemical nematicides with high toxicity and high residue, and the increase in drug resistance, it is particularly important to develop biological nematicides and biological control strategies to control RKN that are environmentally friendly and safe for humans and animals. Owing to the high toxicity of chemicals toward humans and the environment, the use of biocontrol bacteria, a promising method for controlling RKNs, has gained attention. In the prevention and control of plant RKN diseases, biocontrol agents have the advantages of high safety, environmental friendliness, strong specificity, and long control periods (Huang et al., 2015; Liang et al., 2019). At present, our understanding of the interactions between ecosystems, plants, RKNs, and rhizosphere microorganisms at the cellular and molecular levels is limited. Elucidating the molecular mechanism of the interactions between key microorganisms and RKN is of great significance for developing efficient biocontrol agents against plant nematodes and providing new biocontrol strategies for the future control of plant nematodes. Although many nematicidal metabolites from some microorganisms have been identified and some biocontrol bacteria have been developed, there are still many unknown mechanisms involved in biocontrol activity. Therefore, a better understanding of the molecular mechanism of the microbe nematode interaction will provide more effective strategies for further research (Ma et al., 2013; Li et al., 2015). One important aspect is to clarify the “biocontrol bacteria-pathogens-host root microorganisms” interaction, as the relationship is the theoretical basis for improving the colonization and control effect of biocontrol bacteria in the rhizosphere.

Soil is home to the most diverse and abundant microorganisms in the world (Marupakula et al., 2020). Root exudates can “shape” the unique rhizosphere soil bacterial population of plants and have a significant impact on the composition of rhizosphere microorganisms (Zhang et al., 2020a; Liang et al., 2021). Understanding the mutual equilibrium relationship between soil microorganisms and the balance of soil ecosystems is key to preventing the disease from continuing to occur and maintaining the healthy growth of crops (Liu et al., 2016). Microbial imbalances in the “plant–soil–microorganism” ecosystem play an important role in the occurrence of diseases (Shan et al., 2019).

At present, a large number of studies show that the occurrence of plant diseases is closely related to the changes of microbial composition and diversity (Li et al., 2015). The composition and interaction of plant rhizosphere microorganisms and pathogen-associated microorganisms may largely determine the occurrence, development, and spreading of plant diseases and then affect the implementation and effect of biological control. At the same time, the root exudates of different plants at different growth stages are different, which affects the species, quantity, and distribution characteristics of rhizosphere microorganisms. Therefore, this project aims to examine the relationship between tobacco RKNs and rhizosphere microbial populations, analyze the dynamic changes of tobacco rhizosphere bacterial communities and RKNs over various periods of time.

## Materials and Methods

### Sample Collection

Yunyan 97 was used as the experimental flue-cured tobacco variety, and tobacco seedling raising and field management were carried out according to conventional cultivation and management measures (no application of chemicals containing RKNs); the susceptible samples were from the perennial disease site of southern RKNs (2 years in the same plot), and the healthy samples were from a healthy growing tobacco field in the same region (2 years in the same plot). The varieties and cultivation and management measures of the two groups of samples were the same.

BS represents the abbreviation of the soil before transplanting plants, AS represents the soil after tobacco planting and harvesting, and HR0 represents the root of the tobacco seedlings to be transplanted. The flue-cured tobacco seedlings were intensively raised, and the seedlings of the two areas were all raised with the same batch of materials and seeds and in the same period. Therefore, unified sampling was adopted for the tobacco seedlings in the transplanting period. Three seedling trays were randomly selected from the seedling pool, and three seedlings were taken from each tray, which were mixed to form a duplicate sample.

After transplanting, the samples were collected according to different growth stages (15, 30, 45, 60, 75, and 90 days). The field sample collection was performed according to the method described by Peiffer et al. (2013). The roots of three random plants were sampled from the middle of each plot. For each plant, a root segment 5 cm in length and 0.5–3 mm in diameter was collected near the base of the plant, along with any adherent soil particles. The preparation of the collected samples was performed according to the literature of Lundberg et al. (2012). One portion was frozen in a −80°C refrigerator, and the other was used for soil total DNA extraction.

### DNA Extraction, PCR Amplification, and Sequencing

For all soil, rhizosphere and endophytic microbial DNA extractions were performed with the MP Bio Fast DNA Spin Kit for soil (SDS/mechanical lysis; Lundberg et al., 2012). Primers were designed as follows: 515 forward (5′-GTGCCAGCMGCCGCGG-3′) and 806 reverse (5′-GGACTACHVGGGTWTCTAAT-3′) were used to amplify the V3-V4 region of the 16S rDNA gene, and SSU0817 forward (5′-TTAGCATGGAATAATRRAATAGGA-3′) and 1,196 reverse (5′-TCTGGACCTGGTGAGTTTCC-3) were used to amplify the V5-V7 region of the 18S rDNA gene. PCR was carried out with 15 μl of Phusion® High-Fidelity PCR Master Mix (New England Biolabs), 2 μM forward and reverse primers, and approximately 10 ng of template DNA. The thermal cycling process consisted of initial denaturation at 98°C for 1 min, followed by 30 cycles of denaturation at 98°C for 10 s, annealing at 50°C for 30 s, elongation at 72°C for 30 s, and finally, the sample was held at 72°C for 5 min. The library was sequenced on an Illumina NovaSeq platform.

### Bioinformatics and Statistical Analyses

Sequence analysis was performed using Uparse software (Uparse v7.0.1001). Sequences with ≥97% similarity were assigned to the same OTUs. To study the phylogenetic relationships of different OTUs and the differences between the dominant species in different groups, multiple sequence alignment was conducted using MUSCLE software (version 3.8.31). Alpha diversity was applied to analyze the complexity of the species diversity for a sample through three indices, including Chao1, Shannon, and good coverage. All these indices in our samples were calculated with QIIME (version 1.7.0) and visualized with R software (version 2.15.3). The beta diversity based on weighted UniFrac was calculated using QIIME software (version 1.9.1). Random forest analysis evaluates the importance of each predictor by determining how much the mean square error (MSE) increases. The variables were selected when the predictor variables were randomly replaced and the other variables remained unchanged. These analyses were performed using the “RandomForest” package in R. The “Hmisc” package was used to calculate the correlation, the “igraph” package was used to analyses the co-occurrence network (Csardi and Nepusz, 2006), and Gephi 0.9.2 software was used to draw the network map. The statistical analyses using the R software and GraphPad Prism.

### Isolation of Rhizosphere Bacteria

The rhizosphere soil samples of tobacco infected with RKN 15 and 75 days after transplanting were cleaned with sterilized 10 mM MgCl_2_. The cleaned samples were placed into a 2 ml round bottom centrifuge tube, an appropriate number of sterilized metal grinding balls were added, and the sample was ground twice (30 s each time) with a sample grinder. The milled sample was precipitated for 15 min at room temperature; after 5 min, the supernatant was taken for gradient dilution (10^−3^, 10^−4^, 10^−5^, 10^−6^), and the diluted sample was transferred into a 96-well plate with liquid medium (repeated three times for each concentration). After 5 days at a constant temperature of 28°C, the culture medium was drawn and coated in the corresponding solid medium. After culturing at 28°C, plates with fewer than 30 colonies within 10 days were selected, and then, the colonies were marked and purified at −80°C until use.

### Identification of the Isolated Rhizosphere Bacteria at the Molecular Level

A 16S rRNA amplification sequence was used to identify the isolated tobacco rhizosphere bacteria. A single bacterial isolate was cultured in LB medium shaken at 30°C (180 rpm), and the total genomic DNA was extracted from the overnight culture of a single bacterial isolate using a DNeasy® UltraClean® Microbial Kit (QIAGEN GmbH) according to the kit’s instructions. The 16S rRNA gene was amplified by PCR using the universal primers 27F (5′-agagtgtgatcmtggctcag-3′) and 1492r (5′-tacggytaccttgttacgactt-3′). The polymerase chain reaction (50 μl) consisted of 1 μl of bacterial DNA template, 25 μl of 2× Taq PCR master mix, 1 μl each of forward and reverse primers, and 22 μl of ddH_2_O. The PCR conditions were as follows: denaturation at 94°C for 5 min, denaturation at 94°C for 30 s, annealing at 55°C for 30 s, extension at 72°C for 1 min and 30 s, a total of 35 cycles, and extension at 72°C for 10 min. The sequencing results were submitted to the NCBI GenBank database for homology sequence alignment.

### Determination of the Growth-Promoting Activity of the Strains

Screening of phosphate and potassium-dissolving bacteria: the screened strains were inoculated using high-pressure sterilized toothpicks into plates of organic phosphorus, inorganic phosphorus, and a silicate bacteria culture medium and then cultured in a 28°C constant temperature incubator for 7 days to observe whether there were clear and transparent circles and oil drop-like colonies on the plates.

Screening of nitrogen-fixing bacteria: the screened strains were inoculated with disposable inoculation rings on Azotobacter culture medium and observed continuously for 7 days to select the strains with good growth.

Screening of the indole acetic acid (IAA)-producing ability of strains: the classic Salkowski method was used. The activated strains were inoculated in King’s medium containing L-tryptophan and shaken at 30°C and 180 R/min for 2 days. One hundred microliters of bacterial suspension was placed into the wells of white porcelain plates, and then, 100 μl of Salkowski colorimetric solution was added. At the same time, 100 μl of culture medium inoculated with sterile water was added to a Salkowski colorimetric solution as the control. After the white porcelain plate was placed in the dark for 15 min, the control culture medium had no color change, while the culture medium in the hole of white porcelain plate turned red, indicating that the tested strain had the ability to secrete IAA.

Screening of the ability of a strain to produce an iron carrier: the screened strain was inoculated with a sterilized toothpick on the CAS plate, with three points on a plate and three replicates for each strain, and incubated at 30°C for 48 h. An obvious orange halo indicative of an iron carrier appeared around colonies of bacteria secreting an iron carrier. Generally, the larger the circle, the darker the color, and the greater the ability to produce iron carriers.

## Results

### The Diversity of Tobacco Rhizosphere Bacteria Was Reduced by Infection With RKN

**Figure 1:**
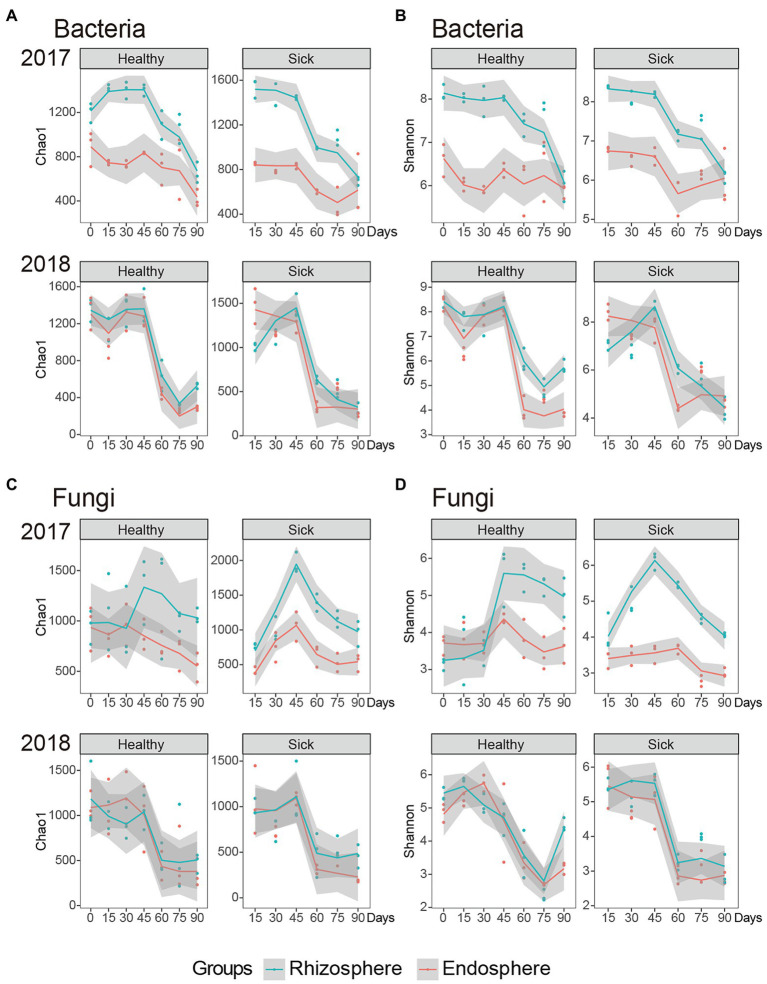
Abundance and diversity of root microorganisms in RKN-infected and healthy tobacco. **(A)** The Chao1 index of rhizosphere and endophytic bacteria. **(B)** The Shannon index of rhizosphere and endophytic bacteria. **(C)** The Chao1 index of rhizosphere and endophytic fungi. **(D)** The Shannon index of rhizosphere and endophytic fungi.

For the alpha diversity analysis, Chao1 estimators and Shannon indices were used to assess the community richness and diversity, respectively. We first analyzed the effect of RKN infection on bacteria. The results indicate that the richness of rhizosphere bacteria infected with RKNs was significantly higher than that of healthy tobacco at the beginning of the first year of the test (Figure 1A). When the planting reached 45 days, the richness of the rhizosphere bacteria of both healthy and diseased plants was significantly reduced, indicating that when the growth process reached 45 days, the abundance of rhizosphere bacteria changed significantly. At the same time, we observed the change trend of Chao1 in the second year of planting, and we found that the rhizosphere microbial abundance of the RKN-infected plants was significantly lower than that of healthy plants at this stage. There was no significant difference between the two at 45 days of planting. However, when the growth cycle reached 45 days, the microbial abundance of healthy and RKN-infected rhizospheres also decreased significantly, indicating that tobacco growth at 45 days may be an important period for the change in rhizosphere microbial abundance (Figure 1A). In general, the abundance of RKN-infected and healthy plants was significantly different at the initial stage, and as the plants grew and developed, the difference between the two would decrease. By 45 days, the microbes in the rhizosphere and endophytes were significantly reduced. Meanwhile, the abundance of rhizosphere bacteria was significantly higher than that of endophytic bacteria.

We also analyzed the Shannon index of each group to explore the trend and law of its diversity. At the beginning of the second year, the diversity index of rhizosphere bacteria in the sick group was significantly lower than that of the healthy group, which indicated that the rhizosphere bacterial diversity was significantly reduced by infection with RKN at this stage, and the overall diversity showed a gradual decreasing trend with time (Figure 1B). The diversity of endophytic bacteria infected with RKN was significantly higher than that of healthy tobacco at the beginning of the first year of planting, and there was no significant decreasing or increasing trend in the overall endophytic bacteria diversity with increasing time. In the second year of continuous planting, when the growth cycle reached 45 days, the diversity of endophytic bacteria decreased significantly. In general, in the first year of planting, the diversity of rhizosphere bacteria in RKN-infected tobacco was not significant, similar to that of healthy tobacco, but the diversity of endophytic bacteria significantly increased. In the second year of infection, the diversity of infected strains was significantly lower than that of healthy plants, and the overall diversity showed a downward trend.

### The Diversity of Tobacco Rhizosphere Fungi Was Increased by Infection With RKN

We analyzed the abundance of rhizosphere and endophytic fungi. The abundance of rhizosphere fungi in the healthy tobacco group did not change significantly in the first year of planting. The abundance of rhizosphere fungi in the RKN-infected group increased gradually within 0–45 days (*p* < 0.01) and began to decrease gradually after 45 days (*p* < 0.01). In the second year of continuous planting, the abundance of rhizosphere fungi in the RKN-infected tobacco group showed no significant change. Similar to the first year, the abundance of rhizosphere fungi was highest at 45 days and then decreased significantly (*p* < 0.05). We further analyzed the abundance of endophytic fungi and found that began to decrease in both the healthy and infected groups after 45 days of the growth cycle in the first year of planting and the second year of continuous planting (*p* < 0.01; Figure 1C). In general, RKN infection significantly increased the abundance of rhizosphere fungi in the early growth stage, which was significantly higher than that in the healthy group, and significantly decreased the fungal abundance in the endophytes at 45 days of growth.

By analyzing the diversity of rhizosphere and endophytic fungi, we found that the diversity of rhizosphere fungi in the RKN-infected group was significantly higher than that in the healthy group in the first year of planting and showed a significant increasing trend in the growth cycle of 0–45 days (*p* < 0.01). At 45 days, the maximum value gradually decreased (*p* < 0.01). In the second year of continuous planting, the diversity of rhizosphere fungi did not change significantly during Days 0–45 but decreased significantly after 45 days (*p* < 0.01). The diversity of endophytic fungi in the RKN-infected group was significantly lower than that in the healthy group after 75 days of the growth cycle (*p* < 0.05) and decreased significantly from 45 days in the second year of continuous planting (*p* < 0.01; Figure 1D).

Two-way ANOVA was conducted to determine the effects of treatment (healthy and sick) and the time period and their interactions on Shannon and Chao1 index (Supplementary Table S1). Through the results, we found that the Shannon and Chao1 index of the bacteria and fungi communities remained relatively stable under treatment in all periods.

### The Effects of RKN Infection on Tobacco Root Microorganisms

**Figure 2:**
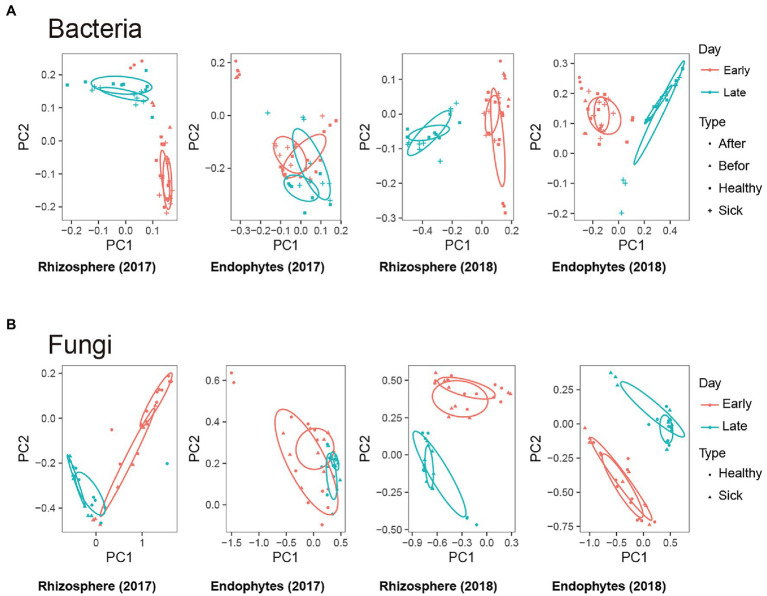
Effects of root-knot nematode (RKN) infection on tobacco root microorganisms. **(A,B)** Representative tobacco rhizosphere bacteria, endophytic bacteria, rhizosphere fungi and endophytic fungi based on a weighted UniFrac algorithm of PCoA analysis. Early: 0–45 days, Late: 45–90 days.

To measure the extent of the similarity between microbial communities, we performed PCoA analysis on rhizosphere microbes and endophytic microbes for each group of samples based on the weighted UniFrac algorithm. We found that whether rhizosphere bacteria or endophytic bacteria (Figure 2A) were present, there were significant differences in the total microbial composition among the groups. Among the PCoA analyses, the PC1 distribution with the largest contribution rate revealed changes of 22.04 and 28.72%, and the PC2 distribution revealed changes of 56.96 and 38.68%, respectively. This result indicates that different growth and development times could significantly change and affect the composition of the tobacco rhizosphere and endophytic bacteria. At the same time, the microbial compositions of the infected and healthy tobacco plants were similar at some time points, and some were significantly different, which indicated that the effects of RKN infection on specific healthy tobacco root microorganisms were different, which may be related to the time of growth and development and the mechanism of infection. The PCoA analysis to compare the similarity of fungal microorganisms in different treatment groups found that the results were similar to those of bacteria (Figure 2B). In summary, the above results showed that RKN infection changed the overall composition of bacteria and fungi in the root system and changed the composition and distribution of root microorganisms in different growth stages of healthy tobacco with increasing time.

### RKN Infection Caused Changes in Bacterial Community Structure at Different Times

**Figure 3:**
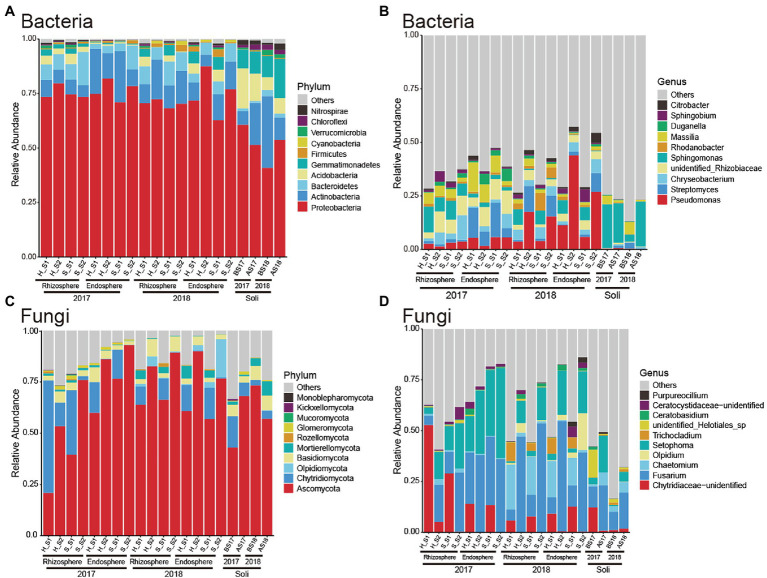
Composition of microorganisms at the phylum and genus levels in RKN-infected and healthy tobacco at different times. **(A)** The relative contributions of the top 10 phyla of bacteria at the phylum level at different times in RKN-infected and healthy tobacco. **(B)** The relative contributions of the top 10 genera of bacteria. **(C)** The relative contributions of the top 10 fungi at the phylum level at different times. **(D)** The relative contributions of the top 10 genera of fungi at the genus level at different times in tobacco roots. S1: 0–45 days, S2: 45–90 days.

To explore the specific distributions in different growth periods and the change rule after RKN infection, we further analyzed the composition of microorganisms at the phylum and genus levels on rhizosphere and endophytic bacteria. We first observed and analyzed the bacteria at the phylum level (Figure 3A). The rhizosphere bacteria were mainly composed of *Proteobacteria*, *Actinobacteria*, *Bacteroidetes*, and *Acidobacteria*. There was no significant difference between the rhizosphere bacteria of the plants infected with RKN and those of healthy tobacco plants at the phylum level, and there was no significant change trend with time. The composition of soil microorganisms was different from that of rhizosphere microorganisms, especially *Gemmatimonadetes*. It was found that the structure of endophytic bacteria was similar to that of the rhizosphere. The results showed that RKN infection did not significantly change the root microbial composition at the phylum level.

We further analyzed the composition of bacteria at the genus level in tobacco and found that they were mainly composed of *Pseudomonas*, *Streptomyces*, *Chryseobacterium*, *Rhizobiaceae*, and *Sphingomonas* (Figure 3B). The relative abundance of *Pseudomonas* increased significantly in the second year compared with the first year. The abundance of *Sphingomonas* in the rhizosphere bacteria was significantly reduced in the early growth period and the abundance of *Rhizobiaceae* increased after RKN infection. RKN infection significantly promoted the proliferation of *Chryseobacterium* when growth reached 3 months. In the first year of planting, the relative abundance of *Streptomyces* of endophytic bacteria decreased significantly in the late growth period, while the relative abundance of *Pseudomonas* increased. The results showed that infection with RKN can change the root microorganisms of healthy tobacco.

### RKN Infection Caused Changes in Fungal Community Structure at Different Times

We analyzed the specific composition of fungal microorganisms in different treatment groups at the phylum level and found that both rhizosphere and intraroot fungi were mainly composed of *Ascomycota* and *Chytridiomycota* (Figure 3C). In the first year of planting, *Chytridiomycota* was the most abundant and dominant microorganism in the rhizosphere and endomycota. With the increase in the growth cycle, the relative abundance of *Chytridiomycota* decreased significantly and that of *Ascomycota* increased, which gradually became the dominant flora. As the growth period increased, the fungal composition was stable, and *Ascomycota* became the main dominant fungus. In the second year of continuous planting, *Ascomycota* and *Chytridiomycota* together formed different groups of fungi in the early growth cycle.

We analyzed the distribution and composition of fungal microorganisms at the genus level (Figure 3D) and found that in the first year of planting, during the early growth cycle, the fungi were mainly composed of *Chytridiaceae*, *Fusarium*, and *Setophoma*, the relative abundance of *Chytridiaceae* gradually decreased, the *Fusarium* abundance gradually increased, and the relative abundance of *Setophoma* also gradually increased, accounting for approximately 10%. At this time, we found that the abundance of *Fusarium* in endophytes was greater than that in the rhizosphere. At the late growth cycle, the abundance of *Chytridiaceae* decreased to a very low level, *Fusarium* and *Setophoma* became the dominant fungi, and the abundance of *Setophoma* increased from 10 to 50%. The fungal compositions in the rhizosphere and endophytes were similar and were composed of *Fusarium* and *Setophoma*, and there was no significant difference in their relative abundance.

### Major Differential Microbial Species in RKN-Infected and Healthy Tobacco Root Microorganisms in Different Growth Stages

**Figure 4:**
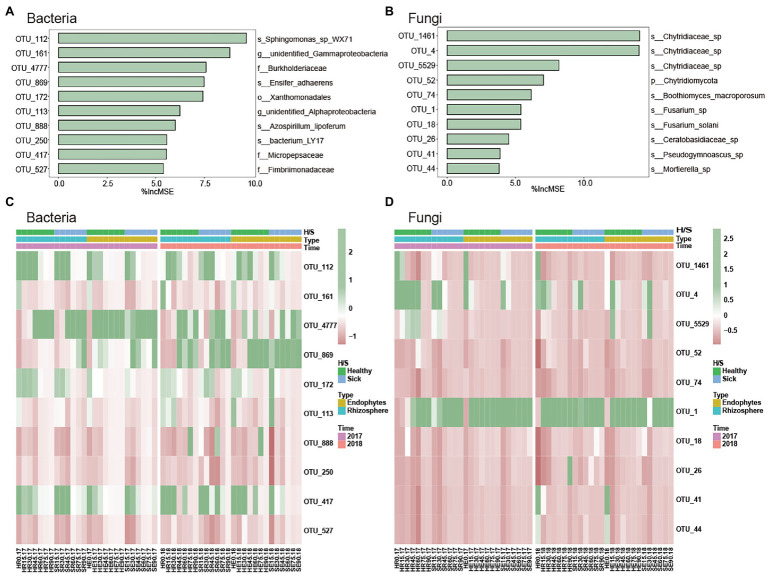
Major differential microbial species in RKN-infected and healthy tobacco root microorganisms in different growth stages. **(A,B)** The top 10 OTUs selected according to %IncMSE through random forest. **(C,D)** Heatmap of the relative abundances of important biomarker microorganisms.

To verify and further determine the microbial OTUs that could be used to discriminate the time of RKN infection, random forest was performed to identify the most important microbial taxa (% Increase MSE) in predicting soil microbes. We selected 10 of the most important time-discriminant OTUs of bacteria and fungi (Figures 4A,B). The results indicated that the most important bacterial phyla taxa were classified *Proteobacteria* and *Armatimonadetes*, and the most important fungi phyla were classified *Chytridiomycota*, *Ascomycota*, *Basidiomycota*, and *Mortierellomycota*. The relative abundances of most of them were low, and they were relatively rare bacterial taxa. At the same time, the abundance of these microorganisms will also change with planting time. For example, the relative abundances of *Sphingomonas*, *Xanthomonadales*, and *Micropepsaceae* were high in the early growth stage of tobacco planting. However, *Burkholderiaceae* was the key group in the late stage of infection (Figure 4C). *Fusarium* and *Chytridiaceae* were the two most abundant fungi. *Chytridiaceae* was the key fungus in the early stage, and the relative abundance of *Fusarium* was high except in the early stages (Figure 4D).

### Comparison of Root Microbial Composition After RKN Infection in Different Planting Years

**Figure 5:**
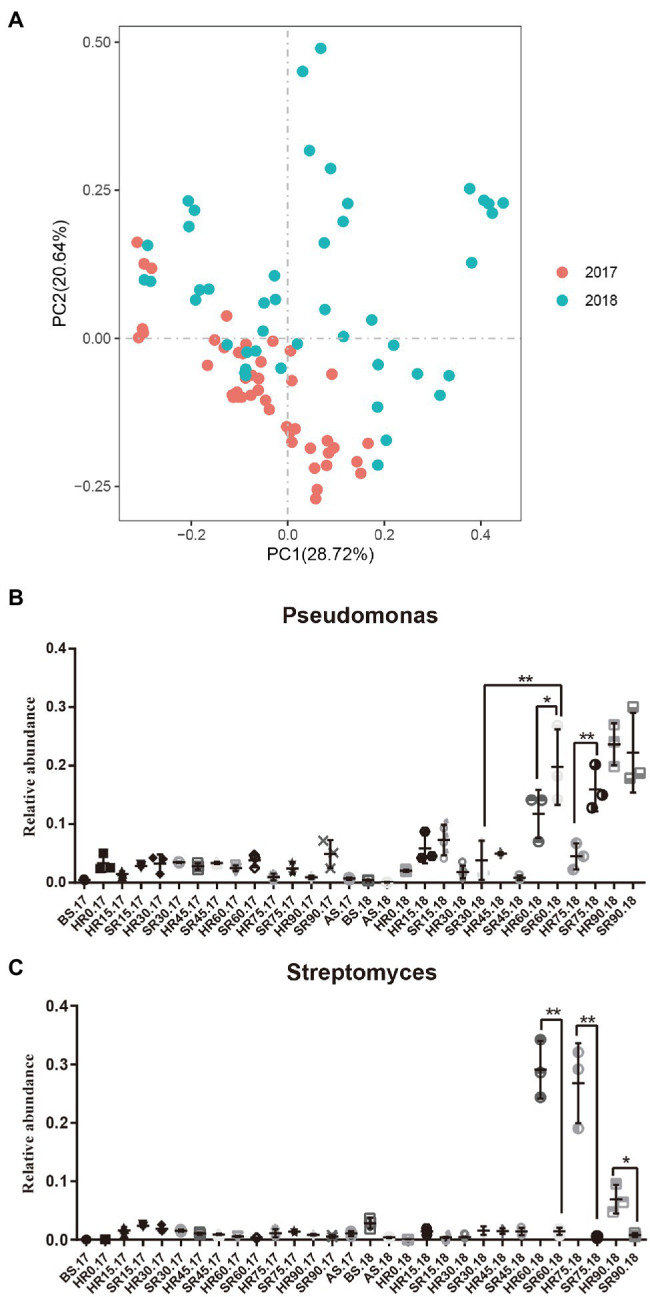
Comparison of root microbial composition after RKN infection in different planting years. **(A)** The distribution and composition of rhizosphere bacteria in 2017 and 2018 analyzed by PCoA based on a weighted UniFrac algorithm. **(B,C)** The relative abundances of *Pseudomonas* and *Streptomyces* in rhizosphere bacteria. Data were analyzed by ANOVA (^*^p < 0.05 and ^**^p < 0.01).

In addition to the analysis of the dynamic changes of root microorganism in different growth periods (0, 15, 30, 45, 60, 75, and 90 days), we also compared and analyzed the root microbial composition in 2017 and 2018 to explore the effect of RKN infection on root microorganisms in different planting years. We found that the root microbes in 2017 were significantly different from those in 2018 (Figure 5A; Supplementary Figures S1A–C). At the same time, we analyzed the rhizosphere bacteria in the second year of continuous planting and found that the relative abundance of *Pseudomonas* in SR60.18 was significantly higher than that in HR60.18 from the 60-day growth cycle (*p* < 0.05, Figure 5B), while the relative abundance of *Streptomyces* was significantly lower than that in HR60.18 (*p* < 0.01, Figure 5C). *Pseudomonas* plays an important role in plant pest control, which also shows that RKN infection from the second year of continuous planting will significantly change the composition of rhizosphere bacteria, especially the proliferation of *Pseudomonas* and the reduction of *Streptomyces*.

### Network Chart of Microbial Flora Based on Co-occurrence

**Figure 6:**
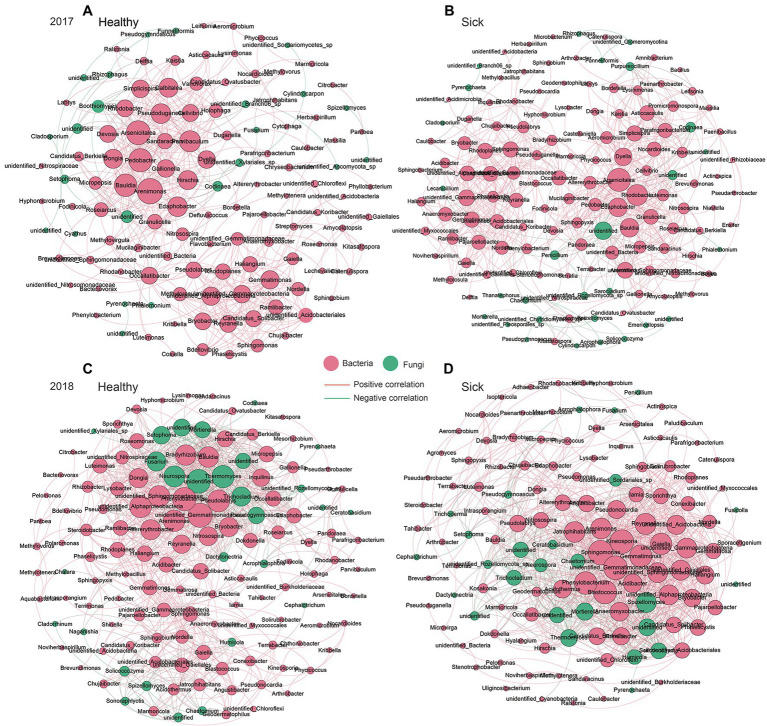
Co-occurrence network relationship between the healthy group and the sick group. **(A)** Healthy tobacco in 2017. **(B)** Sick tobacco in 2017. **(C)** Healthy tobacco in 2018. **(D)** Sick tobacco in 2018. Screen the correlation greater than 0.8, *p* < 0.05. The red edges represent bacteria, and the green edges represent fungi, the red lines between the nodes indicate a positive correlation, and the green lines indicate negative correlation.

Based on the Spearman correlation analysis of relative abundances at the genus level, we analyzed the symbiotic network relationship between the healthy group and the sick group, respectively (Figure 6). The results showed that with the change of planting time from 2017 to 2018, the symbiotic dominant flora in the healthy group changed from *Arsenicitalea*, *Pedobacter*, *Arenimonas*, *Gemmatimonas*, *Bauldia*, etc. to *Neurospora*, *Thermomyces*, *Gemmatimonadacea*, *Alphaproteobacteria*, etc., which as the new dominant flora. It promoted the symbiotic relationship dominated by bacteria to gradually transform into fungi. We also analyzed the symbiotic relationship in the infection group and found that it changed with the planting time from 2017 to 2018, the dominant symbiotic flora after RKN infection gradually changed from *Arsenicitalea*, *Rhodobacter*, etc. to *Sphingomonas*, *Arenimonas*, *Kineosporia*, etc. The above results not only show that RKN infection will change the core flora of the dominant symbiotic relationship, but also show that fungi gradually occupy part of the symbiotic relationship with the increase of planting time.

### Analysis of the Growth-Promoting Characteristics of Strains

According to the abovementioned 16S rRNA sequencing results, 45 different representative strains were selected based on the analysis of the different microorganisms between the RKN-infected group and the healthy group. A total of 32 strains of growth-promoting bacteria were screened from tobacco rhizosphere bacteria infected with RKN (Table 1). Three strains of phosphorite-dissolving bacteria, seven strains of phosphate-solubilizing strains, two potassium-dissolving strains, three nitrogen-fixing strains, 17 IAA-producing strains, and 13 siderophore-producing strains were used. Among them, *Agrobacterium fabrum* YC2096 had the ability to dissolve potassium, IAA, and siderophores; *Pseudomonas* YC2103 had the ability to dissolve phosphorus and iron carriers; *Bacillus* YC2128 had the ability to dissolve phosphorus and produce IAA; and *Enterobacter* YC2132 and *Pantoea agglomerans* YC2174 both had the ability to dissolve phosphorus and produce IAA and siderophores.

**Table 1:** Test results of the growth-promoting activity of the strain.

Serial number	Strain number	Bacteria	Phosphorus dissolving (3)	Phosphorus solution (7)	Nitrogen fixation (3)	Potassium solution (2)	IAA (17)	Iron carried (13)
1	YC2076	*Bacillus oleronius*	−	−	−	−	−	−
2	YC2077	*Devosia riboflavina*	−	−	−	+	−	+
3	YC2083	*Herbaspirillum seropedicae*	−	−	−	−	+	−
4	YC2096	*Agrobacterium fabrum*	−	−	+	−	+	+
5	YC2097	*Rathayibacter*	−	−	−	−	+	+
6	YC2098	*Stenotrophomonas rhizophila*	−	−	−	−	+	−
7	YC2099	*Sphingomonas*	−	−	−	−	−	−
8	YC2100	*Arthrobacter*	−	−	−	−	+	−
9	YC2101	*Bacillus oleronius*	−	−	−	−	−	−
10	YC2102	*Burkholderia paludis*	−	−	−	−	−	−
11	YC2103	*Pseudomonas*	+	+	−	−	−	+
12	YC2109	*Chryseobacterium*	−	−	−	−	+	−
13	YC2111	*Shinella*	−	−	−	−	+	+
14	YC2114	*Agrobacterium*	−	+	−	−	−	+
15	YC2115	*Advenella incenata*	−	−	−	−	−	+
16	YC2116	*Brachybacterium*	−	−	−	−	−	+
17	YC2117	*Ensifer*	−	−	+	−	+	−
18	YC2120	*Stenotrophomonas rhizophila*	−	−	−	−	−	−
19	YC2122	*Microbacterium*	−	−	−	−	+	−
20	YC2124	*Lysobacter capsici*	+	−	−	−	−	−
21	YC2126	*Pedobacter soli*	−	−	−	−	+	+
22	YC2128	*Bacillus*	+	+	−	−	+	−
23	YC2130	*Pectobacterium*	−	−	−	−	+	−
24	YC2132	*Enterobacte*	−	+	−	−	+	+
25	YC2134	*Pectobacterium*	−	+	−	−	+	−
26	YC2136	*Delftia*	−	−	−	−	−	+
27	YC2137	*Alcaligenaceae bacterium*	−	−	−	−	−	+
28	YC2138	*Delftia*	−	−	−	−	−	+
29	YC2139	*Enterobacter*	−	−	−	−	−	−
30	YC2140	*Pseudomonas*	−	−	−	−	−	+
31	YC2141	*Agrobacterium*	−	−	+	−	+	+
32	YC2143	*Stenotrophomonas rhizophila*	−	−	−	−	−	+
33	YC2145	*Pseudomonas punonensis*	−	−	−	−	−	−
34	YC2147	*Erwinia*	−	−	−	−	−	−
35	YC2149	*Chryseobacterium*	−	−	−	−	+	−
36	YC2151	*Variovorax paradoxus*	−	−	−	−	−	−
37	YC2153	*Arthrobacter nitrophenolicus*	−	−	−	+	−	+
38	YC2155	*Pseudomonas putida*	−	−	−	−	−	−
39	YC2157	*Alcaligenaceae bacterium*	−	−	−	−	−	+
40	YC2159	*Alcaligenes*	−	−	−	−	−	+
41	YC2165	*Rothia marina*	−	−	−	−	−	−
42	YC2167	*Janibacter melonis*	−	−	−	−	−	−
43	YC2169	*Bacterium*	−	−	−	−	−	−
44	YC2171	*Exiguobacterium acetylicum*	−	+	−	−	−	+
45	YC2174	*Pantoea agglomerans*	−	+	−	−	+	+

In recent years, it has been found that the bacteria and metabolic pathways producing IAA and iron carriers are significantly related to nematode infection in plants. Studies on the microbiome and biocontrol have found that strains and mixtures with these functions have more research and application prospects in biocontrol. Therefore, a combination of 16S rRNA sequencing and life-promoting tests was used to screen a variety of strains with high biological activity, which has important application value in controlling RKN infection and promoting plant growth and development and provides strain resources and a new reference basis for the development of biologically controlled bacteria.

## Discussion

At present, biocontrol microorganisms are an important resource for the development of nematode biocontrol preparations (Dong et al., 2018). Nematophagous fungi kill nematodes through predation, parasitism, and toxin production. They are an important group of microorganisms that control nematode populations in nature; nematophagous bacteria are another important resource for the biocontrol of nematodes. The bionematocides developed using nematode-preying fungi and bacteria have been registered and used to control nematode disease in plants. Therefore, the development of biologically controlled bacteria has important significance for and effects on the treatment of RKN. This experiment explored the effects of different growth periods (0, 15, 30, 45, 60, 75, and 90 days) and continuous planting on the root system microorganisms (rhizosphere and endophytic) of healthy tobacco after infection with RKN through 16S and 18S rRNA technology. We found that in the first year of planting, the diversity of rhizosphere bacteria in RKN-infected tobacco was not significant, similar to that of healthy tobacco, but it significantly increased the diversity of endophytic bacteria. In the second year of infection, the diversity of infected strains was significantly lower than that of healthy plants, and the overall diversity showed a downward trend. Therefore, by comparing the composition and diversity of rhizosphere bacteria between infected and healthy tobacco, we found that there were some differences in the α-diversity and distribution of rhizosphere bacteria between diseased and healthy tobacco, indicating that RKN infection affected the rhizosphere bacterial flora of host plants to a certain extent.

We found that RKN infection significantly changed and affected the composition and abundance of rhizosphere and endophytic bacteria in tobacco. At the same time, the composition of root microorganisms changed significantly with the time of different growth stages. RKNs significantly reduced the abundance of *Sphingomonas*, increased the abundance of *Rhizobiaceae* in the early growth stage of the first year of planting, and promoted the proliferation of *Chryseobacterium* during the late growth stage. From 60 days after the second year of planting, the RKNs significantly promoted the proliferation of *Pseudomonas* and reduced the abundance of *Streptomyces*. In the first year of planting of the endophytic bacteria, the relative abundance of *Streptomyces* decreased significantly, while the relative abundance of *Pseudomonas* increased. In the second year of planting, the abundance of *Pseudomonas* increased from 60 days of growth. Therefore, we found that both rhizosphere and endophytic bacteria showed significant regular change trends with different growth stages, and the microorganisms compositions were different. Infection with RKN can partially change the root microorganisms of healthy tobacco. On the 90th day of the second year of continuous planting, the relative abundance of *Bacillaceae* in the RKN group was significantly higher than that in the healthy group. In the “plant-pathogen-beneficial bacteria” interaction system, the plant root exudes nutrients and exogenous signaling substances, thereby affecting the behavior of rhizosphere bacteria, and selecting beneficial microbial populations to resist pathogen infection has been confirmed and widely accepted (Buchan et al., 2010). RKNs infect plants, leading to host physiological changes. Nutrients and metabolites are released through root cells fed by nematodes through the symplast, which changes the composition of root exudates, such as water-soluble carbon and metal ions (Tian et al., 2015), and then affects the composition of rhizosphere bacteria. Rhizosphere bacteria play key roles in plant growth, health, and development and are mainly determined by the soil community. To some extent, they are regulated by the biological clock, related to plant metabolism, and affect carbon metabolism and the exchange of plant microorganisms (Staley et al., 2017). Mavrodi et al. (2012) found that the population of *Pseudomonas* spp. producing DAPG was higher in the rhizosphere soil of wheat cultivated in paddy fields dominated by *Gaeumannomyces graminis*, while in the same region, the number of *Pseudomonas* spp. producing the resistance metabolite phenazine was higher in the rhizosphere soil of early wheat with sheath blight. The results showed that there was no significant difference in the soil microbial population structure between inoculated and uninoculated barley, but the overall decline phenomenon only occurred in the soil inoculated with the pathogen (Schreiner et al., 2010). This conclusion was consistent with our findings. We found that the relative quantities of *Bacillaceae* and *Pseudomonas* increased significantly after RKN infection with increasing growth time, and RKN infection caused a large number of proliferations of *Pseudomonas* in the second year of continuous tobacco planting. *Pseudomonas* bacteria have been widely reported to inhibit the activity of RKN by various mechanisms, which is beneficial for inhibiting soil-borne diseases and providing a healthy soil environment to promote root growth (Li et al., 2015; Nikolić et al., 2019). Tao et al. (2020) conducted a high-throughput screening of 886 rhizosphere samples of 886 plants in China’s three major cotton-producing areas and found that two *Pseudomonas* strains showed significant inhibitory effects on Vibrio dahlia. Through comparative genomic and phenotypic analyses, it was shown that *P. protegens* XY2F4 and *P. donghuensis* 22G5 were the most effective strains to protect cotton plants against verticillium wilt because they produced specific biological control products. In addition, they found and confirmed that the natural tropolone compound 7-hydroxytropolone (7-HT) had a significant effect on the general public. Research has revealed that *Pseudomonas* bacteria have a specific gene cluster that can produce effective antipathogenic metabolites, which can now be used as new drugs for the biological control of Verticillium wilt. Continuous cropping leads to obstacles in crop productivity by the accumulation of p-hydroxybenzoic acid (PHBA) and ferulic acid (FA). Zhang et al. (2020b) used transcriptomics to explore the mechanism by which a strain of *Pseudomonas* CFA has higher PHBA and FA degradation abilities in soil, proposed a complete pathway for the conversion of PHBA and FA to acetyl-CoA, and discovered that 4-hydroxybenzoate 3-monooxygenase and vanillate O-demethylase were rate-limiting enzymes by gene overexpression. A previous study showed that *Pseudomonas* CFA has the potential to alleviate the PHBA and FA stress of cucumber and alleviate continuous cropping obstacles. The above results all indicate that plant growth-promoting rhizobacteria are efficient candidates for application in agricultural fields to enhance crop yield and suppress plant diseases. However, a wide variety of root exudates enter the rhizosphere soil after RKN infection. Some of these root exudates can directly antagonize diseases and insect pests, while others act as signal substances to gather and select microbial flora conducive to plant growth under the action of microbial chemotaxis (Bücking et al., 2008). The above results showed that beneficial microorganisms could be selected specifically to resist pathogen infection. That is, RKN infection could affect the behavior of rhizosphere bacteria and select some beneficial microbial populations to resist pathogen infection. The above results indicate the mechanism by which *Pseudomonas* regulates and inhibits pests and diseases, suggesting that in this experiment, tobacco infected with RKN can promote the proliferation of Pseudomonas and play a biological control function.

In addition, comparing the results of root microbes in 2017 and 2018, we found that in the second year of continuous tobacco planting, RKN infection significantly reduced the abundance of *Streptomyces* in both the rhizosphere and endophytes. *Streptomyces* is a main plant rhizosphere growth-promoting bacterium and an important nematode biocontrol bacterium. The bioactive substances produced by *Streptomyces*, such as antibiotics, enzymes, and inhibitors, have good inhibitory effects on RKNs and pathogens. In this study, in the second year of planting, the relative abundance of *Streptomyces* in rhizosphere soil infected by RKN and that in healthy tobacco were significantly different. The abundance of *Streptomyces* in healthy tobacco was significantly higher than that in the rhizosphere after RKN infection, indicating that the composition and abundance of *Streptomyces* may be related to RKN infection. The above results not only reveal that the root microorganisms after 2 consecutive years of planting have significantly different trends but also suggest that *Streptomyces* may be closely related to RKN infection, and subsequent studies can be conducted to define the mechanism of interaction between *Pseudomonas* and *Streptococcus* and RKN.

In this study, 18S rRNA sequencing technology was also used to study the composition of fungal microorganisms. Rhizosphere and endophytic fungi are widely distributed in almost all plant groups and are important microbial plant symbionts. They grow in plant cells or within cells, obtain nutrition from host plants and survive for long periods of time. Their main functions are to promote plant growth, improve the ecological adaptability of host plants, dilute active secondary metabolites, and promote plant restoration. The mechanism by which endophytic fungi promote plant growth is multifaceted. They can assist host plants in absorbing iron ions from soil through biological nitrogen fixation, synthesize, or promote plants to synthesize a variety of plant growth hormones, promote the growth of host roots and the absorption of a variety of inorganic ions, and synthesize some small molecular substances or enzymes to improve the host plants’ resistance to frost and other harmful environmental conditions and the sensitivity of harmful pathogens to promote plant growth. In addition, endophytic fungi can also improve host resistance to fungal diseases. Some studies have also found that endophytic fungal infection can affect the soil microbial community. We found that *Fusarium* and *Setophoma* were the main dominant flora of RKN-infected and healthy tobacco plants in the second year of continuous planting, and the RKN infection group significantly increased the relative abundance of *Setophoma*. The results of this study can provide some theoretical and technical means for the biological control of knot nematode disease.

In this experiment, a combination of 16S rRNA sequencing and a life-promoting test was used to comprehensively screen out strains with a variety of high biological activities. Thirty-two strains of growth-promoting bacteria were screened from tobacco rhizosphere bacteria. At present, *Rhizobia*, *Pseudomonas fluorescens*, and *Bacillus* have been found to have the potential for disease prevention and growth promotion. The growth-promoting mechanism of the abovementioned rhizosphere bacteria for plants mainly includes the synthesis of substances that directly promote the growth and development of plants, such as IAA, which change the form of elements to promote the absorption of nutrients (such as nitrogen fixation and phosphorus solubilization). However, most rhizosphere growth-promoting bacteria have a single function in overcoming nutrient and disease adversity. Therefore, it is very important to screen growth-promoting strains with complex abilities in this experiment to improve rhizosphere nutrition and regulate the soil microecological environment. For example, the *Pseudomonas* found in this study has the compound functions of phosphate solubilization and iron carriage. Iron is one of the essential micronutrients in plants and an indispensable element in life activities. Studies have shown that some biocontrol bacteria can form a competitive iron ion relationship with pathogenic bacteria by producing iron carriers to achieve an inhibitory effect on pathogenic bacteria (Saha et al., 2016). It can also reduce trivalent iron ions, which are not easily absorbed by plants, to bivalent iron ions, which are easily absorbed by plant cells by secreting and producing iron carriers, to improve the iron absorption efficiency of plants and promote the growth of plants. Manwar et al. (2004) found that *Pseudomonas aeruginosa* could increase the germination rate and chlorophyll content of peanut plants and promote root bifurcation and nodule formation. This study provides strain resources and a reference basis for the development of biocontrol and growth-promoting bacteria.

## Conclusion

This study used 16S and 18S rRNA sequencing technology to explore the microbial composition and diversity of tobacco roots at different growth stages in typical tobacco RKN-infected areas for 2 consecutive years to analyze the dynamic changes in the rhizosphere and endophytic microbial communities in tobacco and significantly associated the microbiota of RKN on a time scale. We discovered the overall change trend of tobacco rhizosphere and root endophytic microorganisms with different microorganism growth periods and found that in the “Biocontrol bacteria-pathogens-host root microorganisms” interaction system, infection with RKN after continuous planting will significantly promote the proliferation of *Pseudomonas*, affect the composition of rhizosphere microorganisms, and make the rhizosphere select beneficial microbial populations to resist pathogen infection. This research provides reference data for elucidating the pathogenesis of RKNs and provides new ideas for the biological control of RKNs.

## Data Availability Statement

The datasets presented in this study can be found in online repositories. The names of the repository/repositories and accession number(s) can be found at: https://www.ncbi.nlm.nih.gov/bioproject/PRJNA694781.

## Author Contributions

YC contributed to the conception of the study and drafted the manuscript. Z-XY, D-MY, and NL performed the experiment and manuscript preparation. S-ZY performed the data analyses. J-YM and X-JC helped perform the analysis with constructive discussions and revisions of text passages. All authors contributed to the article and approved the submitted version.

## Funding

The work was supported by the National Natural Science Foundation of China (No. 31660544), the Science and Technology Program of Guizhou Tobacco Company (2021XM12), the Major Science and Technology Program of China Tobacco Corporation [110202101055(LS-15)], the Guizhou Academician Workstation of Microbe and Health [Talent Platform (2020)4004], and the Guizhou Science and Technology Project [(2020)1Y106].

## Conflict of Interest

The authors declare that the research was conducted in the absence of any commercial or financial relationships that could be construed as a potential conflict of interest.

## Publisher’s Note

All claims expressed in this article are solely those of the authors and do not necessarily represent those of their affiliated organizations, or those of the publisher, the editors and the reviewers. Any product that may be evaluated in this article, or claim that may be made by its manufacturer, is not guaranteed or endorsed by the publisher.
