# Supplementary material for: Tobacco Root Microbial Community Composition Significantly Associated With Root-Knot Nematode Infections: Dynamic Changes in Microbiota and Growth Stage
Source: Front Microbiol. 2022 Feb 9;13:807057. doi: 10.3389/fmicb.2022.807057 (PMC8863970; doi:10.3389/fmicb.2022.807057)
Supplement: Supplementary file 1 [file Data_Sheet_1.docx]

Table S1 Mean values±standard deviation of Shannon and Chao1 and results of the two-way ANOVA with treatment (healthy and sick) and growth period as factors.

|  |  | 2017 | | 2018 | | treatment | period | Treatment x period |
| --- | --- | --- | --- | --- | --- | --- | --- | --- |
|  |  | healthy | sick | healthy | sick |  |  |  |
| rhizosphere bacteria | Shannon | 7.59±0.76 | 7.55±0.82 | 6.97±1.40 | 6.43±1.38 | ns | ** | ns |
|  | chao1 | 1178.09±275.70 | 1198.74±313.02 | 964.22±442.28 | 839.04±411.38 | ns | ** | ns |
| endophytic bacteria | Shannon | 6.18±0.44 | 6.28±0.52 | 6.14±1.93 | 6.44±1.60 | ns | ns | ns |
|  | chao1 | 723.09±184.11 | 697.05±175.47 | 839.06±486.68 | 839.87±508.43 | ns | ns | ns |
| rhizosphere  fungi | Shannon | 4.54±1.10 | 4.87±0.80 | 4.49±1.17 | 4.40±1.11 | ns | ns | ns |
|  | chao1 | 1091.02±310.26 | 1205.07±410.06 | 814.33±351.36 | 725.63±305.78 | ns | *** | ns |
| endophytic fungi | Shannon | 3.96±1.04 | 3.32±0.38 | 4.04±0.82 | 4.01±1.23 | ns | ** | ns |
|  | chao1 | 735.72±354.04 | 656.20±255.63 | 859.33±273.74 | 629.84±383.28 | * | ns | ns |

Signif. codes: 0 ‘***’ 0.001 ‘**’ 0.01 ‘*’ 0.05 ‘.’ 0.1 ‘ ’ 1, ns., not significant.


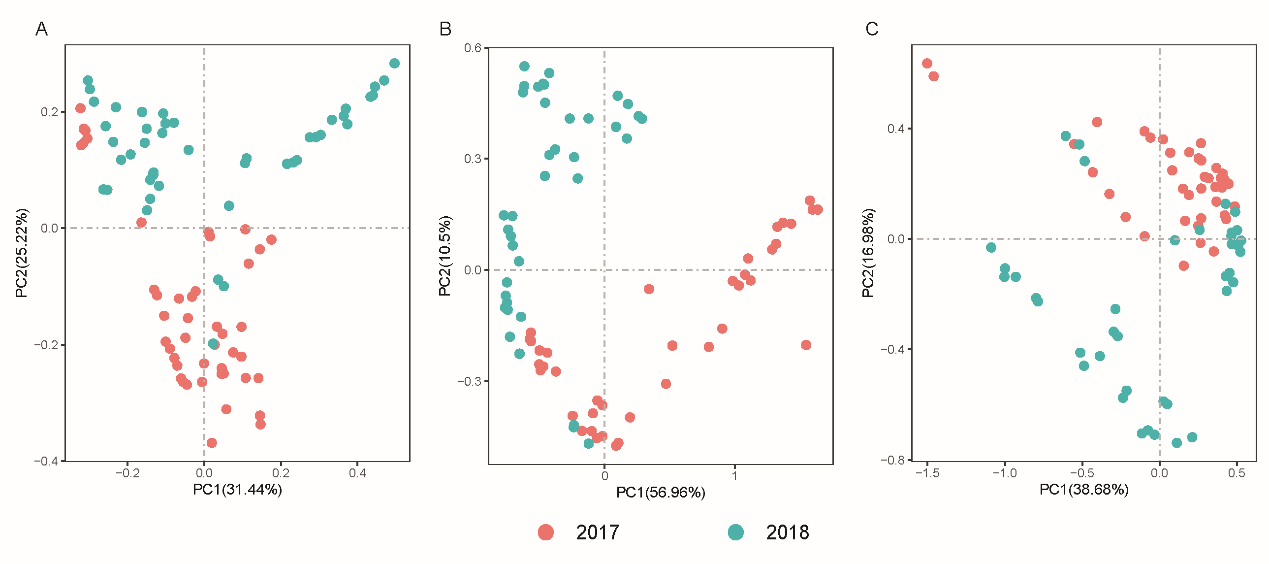


Supplementary Figure S1 A. The distribution and composition of endophytic bacteria in 2017 and 2018 analyzed by PCoA based on weighted unifracs algorithm. B. The distribution and composition of rhizosphere fungi in 2017 and 2018 analyzed by PCoA based on weighted unifracs algorithm. C. The distribution and composition of endophytic fungi in 2017 and 2018 analyzed by PCoA based on weighted unifracs algorithm.
